# Effects of Fungal Solid-State Fermentation on the Profile of Phenolic Compounds and on the Nutritional Properties of Grape Pomace

**DOI:** 10.3390/microorganisms12071310

**Published:** 2024-06-27

**Authors:** Gordana Šelo, Mirela Planinić, Marina Tišma, Ana-Marija Klarić, Ana Bucić-Kojić

**Affiliations:** Faculty of Food Technology Osijek, Josip Juraj Strossmayer University of Osijek, F. Kuhača 18, HR-31 000 Osijek, Croatia; gselo@ptfos.hr (G.Š.); mplanini@ptfos.hr (M.P.); mtisma@ptfos.hr (M.T.);

**Keywords:** grape pomace, phenolic compounds, nutritional composition, enzymes, solid-state fermentation, *Trametes versicolor*

## Abstract

Grape pomace (GP) is considered a natural source of bioactive compounds. To improve the extractability of bioactive compounds, in this work, GP was biologically treated for 15 days with the white-rot fungus *Trametes versicolor* in laboratory jars and a tray bioreactor under solid-state fermentation (SSF) conditions. During SSF, the activity of lignolytic (laccase and manganese peroxidase) and hydrolytic (xylanase, cellulase, *β*-glucosidase, and invertase) enzymes was measured, with the activities of laccase (2.66 U/g_db_ in jars and 0.96 U/g_db_ in the bioreactor) and xylanase (346.04 U/g_db_ in jars and 200.65 U/g_db_ in the bioreactor) being the highest. The effect of the complex enzyme system was reflected in the changes in the chemical composition of GP with increasing ash, crude protein, and free fat content: 28%, 10%, and 17% in the laboratory jars, and 29%, 11%, and 7% in the bioreactor, respectively. In addition, the biological treatment improved the extractability of 13 individual phenolic compounds. Therefore, the applied SSF technique represents an effective strategy to improve the profile of phenolic compounds and the nutritional composition of GP, promoting their valorization and opening the door for potential applications in the food industry and other sectors.

## 1. Introduction

Grapes are produced intensively worldwide, according to the data for 2022 about 75 million tons per year, of which 37% of grapes are produced in Europe [1]. The majority of grapes (almost 80%) is used for wine or juice processing [2]. Therefore, the juice and wine industry produces the largest amount of waste, the so-called grape pomace (GP), which consists of the pulp, skin, seeds, and sometimes stems [3,4]. About 20–30% of the total grapes used for winemaking are left behind in the form of GP [5,6]. According to its chemical composition, GP is rich in proteins, fibers, vitamins, sugars, minerals, etc., and what makes it particularly valuable are the phenolic compounds distributed among flavonoids, such as flavanols, flavonols, anthocyanins, isoflavones, etc.; phenolic acids, such as hydroxycinnamic acid and hydroxybenzoic acid derivatives; and stilbenes, such as viniferin and resveratrol [7,8,9,10]. The mentioned components have antioxidant, anti-inflammatory, cardiovascular, anticancer, cytotoxic, and antimicrobial properties, which is why they have various benefits for human health [5,8,10]. However, not all grape-derived phenolic compounds are extracted during wine production, so about 70% of these bioactive compounds remains in the GP [3]. In order to utilize the bioactive compounds and their benefits, many researchers deal with finding alternative uses of GP, such as obtaining extracts with antioxidant properties, composting and use as biomass for energy production, use in the sustainable production of food enriched with polyphenols and fiber, use in encapsulation techniques, use as fermentation substrates, and feed for ruminants [6,8,10,11,12].

In addition to the fact that a large proportion of phenolic compounds remain in GP after winemaking, it is characteristic not only of GP but of all plant matrices that some phenols are covalently bound to cellulose, hemicellulose, lignin, pectin, and structural proteins in the lignocellulosic structure, which gives them the properties of insolubility and low extractability. Numerous methods are used for the degradation of lignocellulosic biomass and for the extraction of bioactive compounds, but one biological technique that is attracting increasing attention and appears promising for modifying the lignocellulosic complex and releasing soluble phenols is solid-state fermentation (SSF) [11]. SSF is the cultivation of microorganisms on a solid substrate without the presence of free water and offers an alternative for the production of various enzymes, bioactive compounds, and other valuable components at low production costs [13,14]. SSF is a very demanding process whose development is influenced by numerous agents, such as the concentration of the inoculum, the chemical composition of the substrate, pH value, temperature, moisture content, and aeration [15]. Microorganisms from the group of yeasts, bacteria, and fungi are used in SSF, but the most common are filamentous fungi, such as *T. versicolor*, which has aroused great interest in medicine, pharmacy, and food industries since ancient times due to its nutritional and therapeutic properties [13,16].

This study aimed to evaluate the effect of SSF with *T. versicolor* on the release of phenolic compounds from GP and on the modification of the chemical composition of GP at the laboratory scale (jars and tray bioreactor). This work builds on the deficit of literature data on the chemical composition of GP biologically treated with *T. versicolor*, as the novelty of this work is its valuable insights into the modified chemical composition and release of individual phenolic compounds of biologically treated GP by *T. versicolor* under SSF conditions, as well as considerations for its potential application in the pharmaceutical, food, and feed industries.

## 2. Materials and Methods

### 2.1. Chemicals and Reagents

Standards for Ultra-High-Performance Liquid Chromatography (UHPLC) analysis of phenolic compounds (phenolic acids, flavan-3-ols, flavonols, procyanidin, and stilbenes) were obtained from Sigma Aldrich (Saint Louis, MO, USA), Acros Organics (Geel, Belgium), Extrasynthese (Genay, France), and Applihem (Darmstadt, Germany). Reagents for spectrophotometric determination of total phenolic compounds, total flavonoids, and total extractable proanthocyanidins were purchased from Alfa Aesar GmbH & Co KG (Kandel, Germany) and Acros Organics (Geel, Belgium). Standards for UHPLC analysis of sugars were purchased from Acros Organics (Geel, Belgium). UHPLC-grade reagents (methanol, acetonitrile, and glacial acetic acid) were purchased from J.T. Baker (Arnhem, The Netherlands), Macron Fine Chemicals (Gliwice, Poland), and Fisher Chemical (Loughborough, UK). Hydrochloric acid, sulfuric acid, n-butanol, and n-hexane were obtained from Carlo Erba Reagents GmbH (Emmendingen, Germany), and water was deionized in a Milli-Q water purification system (Millipore, Bedford, MA, USA). Ergosterol, 2,2′-azino-bis(3-ethylbenzothiazoline-6-sulfonic acid) diammonium salt (ABTS) and p-nitrophenyl were obtained from Sigma Aldrich (Saint Louis, MO, USA); copper sulfate, 3,5-dinitrosalicylic acid (DNS), p-nitrophenyl-β-D-glucopyranoside, veratryl alcohol, and potato dextrose agar from Biolife Italiana (Milan, Italy); and wheat arabinoxylan from Megazyme Ltd. (Bray, Ireland).

### 2.2. Substrate and Microorganism

The GP sample of Cabernet Sauvignon was provided by a local winery (Erdut) in eastern Croatia. The GP contained pulp, seeds, and skin, and was stored at −20 °C until it was used as a substrate for fungal treatment.

The fungal treatment of GP was carried out with the filamentous fungus *Trametes versicolor* TV6 (Microbial Culture Collection of the National Institute of Chemistry, Ljubljana, Slovenia) cultivated on potato dextrose agar (PDA) at 27 °C for ten days.

### 2.3. Biological Treatment of Grape Pomace by Trametes Versicolor

#### 2.3.1. Laboratory Jars

GP was thawed and coarsely ground to a particle size of about 2–5 mm with a blender (Philips, HR 2860). Then 50 g of GP was mixed with distilled water (30 mL), autoclaved at 121 °C for 15 min, and chilled to ambient temperature. The spore suspension for the inoculation of GP was prepared by vortexing 5 mycelial discs (diameter of 1 cm) of the culture from a Petri dish in 10 mL of sterile water. The spore concentration in the prepared suspensions was 1 × 10^6^ spores/mL. The moisture content of the inoculated GP was approximately 67%. The inoculated substrate in the jars was incubated for 15 days at 27 °C with an adjusted aeration of 10% in an incubator (KB 115, BINDER GmbH, Tuttlingen, Germany). The substrate layer was about 4 cm high. The same procedure was used to prepare the control sample, corresponding to day “0”, with distilled water added instead of the spore suspension.

#### 2.3.2. Tray Bioreactor

SSF in the tray bioreactor was performed by spreading 1150 g of pre-sterilized GP mixed with 150 mL of water on a plate in a tray chamber and cooling to 27 °C after the sterilization of the bioreactor. The inoculation of GP was performed with 25% of the prepared inoculum in relation to the total amount of material on the plate. The inoculum was lightly mixed with the substrate. The inoculum was an already-grown culture of *T. versicolor* in jars for 10 days, according to the procedure described in Section 2.3.1. The inoculated substrate (2 cm high) had a moisture content of about 71%. SSF was carried out under natural aeration without mixing. Sterile water from an external tank connected to the bioreactor was added for humidification. SSF was carried out for 15 days. A schematic representation and details about the tray bioreactor can be found in the paper published by Planinić et al. [17].

After SSF in jars and in the tray bioreactor, the samples were sterilized to stop the SSF process and then dried for 48 hours at ambient temperature, milled to a particle size of ≤1 mm with an ultracentrifugal mill (Retsch ZM200, Haan, Germany), and saved at 4 °C until further analysis.

### 2.4. Enzyme Activity Measurement

For the measurement of enzyme activity after 1–10 and 15 days of fermentation, samples were prepared by extracting 2 g of non-sterilized, biologically treated GP in a buffer solution. The different types of buffer solutions used to prepare the GP extracts for enzyme activity measurement are listed in Table 1. Extraction was conducted by vortexing, shaking the samples for 15 s every 5 min (30 min in total). Samples were then centrifuged at 10,000× *g* for 5 min and the resulting supernatant was used to measure the activity of hydrolytic enzymes (xylanase, cellulase, *β*-glucosidase, and invertase) and lignolytic enzymes (laccase and manganese peroxidase (MnP)). Measurements were conducted in triplicate using a UV–VIS spectrophohtometer (UV-1280, Shimadzu, Kyoto, Japan), and the results are expressed in U/g_db_.

The activities of xylanase (endo-1,4-β-xylanase) and cellulase (endoglucanases and exoglucanases) were determined by the DNS method [18,19], *β*-glucosidase activity was determined according to the study of Karpe et al. [20], and invertase activity was determined by the method of Margetić and Vujčić [21]. The activities of MnP and laccase were determined by monitoring the oxidation of the substrate 2,6-dimethoxyphenol (DMP) at 469 nm for 120 s according to the method of Lueangjaroenkit et al. [22].

### 2.5. Determination of Biomass Concentration

The biomass concentration was measured following the study of Barreira et al. [23] with some modifications, where an indirect method by determining the ergosterol concentration was used.

The methodology for determining the ergosterol concentration includes the method for determining the fat content, after which a saponification was carried out. Two mL of 0.1 M ascorbic acid and ten mL of 2 M potassium hydroxide were added to the flask containing the extract obtained after the extraction of fat in the GP samples. The flasks were placed in a water bath at 60 °C and 125 rpm for 45 min. The contents of the flask were then chilled to ambient temperature and filtered into test tubes. Then, 2.5 mL of sodium chloride (1.7 M) and 5 mL of n-hexane were added to the tubes and subsequently shaken for 1 min. The contents of the test tubes were left on the stand for about 1 min, during which time the water layer and the n-hexane layer containing the sterols separated, then the n-hexane layer was separated into another test tube using a pipette. The extraction procedure of the aqueous layer was repeated two more times with the addition of a new aliquot of 5 mL of n-hexane. All fractions of n-hexane were picked in the tube and a small amount of sodium sulfate was added to dry the remaining water. The contents of the test tube were vortexed, filtered into an evaporating flask, and evaporated at 40 °C in a rotary evaporator (Büchi B-210, Flawil, Switzerland). After evaporation, the contents of the flask were dissolved by adding 2 mL of methanol, filtered through a 0.45 µm porous membrane (Chromafil Xtra PTFE) and used for UHPLC analysis.

The UHPLC analysis involved the use of a PDA detector, recording the spectrum at 280 nm, using a Shim-Pack GIST C18 column (250 × 4.6 mm, 3 μm, Shimadzu, Kyoto, Japan). The conditions of the method were methanol as a mobile phase, injection volume of 20 µL, and flow rate of 1 mL/min, at 25 °C for 20 min. The data were processed in the LabSolutions program (version 5.87), and the mass concentration of ergosterol (mg/L) was expressed per gram of fat (*C*_ERG_, mg/g_F_).

### 2.6. Analysis of Chemical Composition of Grape Pomace

The procedure for the preparation of liquid extracts for chemical composition analyses are shown in Table 2, as well as analyses carried out on solid samples of GP.

#### 2.6.1. UHPLC Analysis of Individual Phenolic Compounds

The UHPLC method with a photodiode array detector (PDA) was used to qualitatively and quantitatively analyze the phenolic compounds in GP liquid extracts, as described in a previously published study [24]. A reversed-phase Kinetex^®^ C18 Coreshell column (100 × 4.6 mm, 2.6 µm, Phenomenex, Torrance, CA, USA) was used for the separation, with a linear gradient of two solvents: solvent A (1.0% acetic acid in water, *v*/*v*) and solvent B (50% methanol, 50% acetonitrile, *v*/*v*). A linear gradient was performed at 30 °C, while maintaining a flow rate of 1 mL/min and injecting a volume of 20 µL of the sample.

#### 2.6.2. Analyses of Total Phenolic Compounds, Total Flavonoids, and Total Extractable Proanthocyanidins

TP was measured using the Folin–Ciocalteu colorimetric method [25]. A calibration curve was prepared with gallic acid. For the TP measurement, an amount of 40 µL of the extract was added to tubes containing 3160 µL of water, and then 200 µL of the Folin–Ciocalteu reagent was added. After 8 min, 600 µL of 20% (*w*/*v*) Na_2_CO_3_ was added. The contents of the tubes were incubated at 40 °C for 30 min. The absorbance was measured at 765 nm compared to a blank.

TFs were measured using the spectrophotometric method with aluminum chloride [26]. A volume of 500 µL of the extract was mixed with 2000 µL of water. Then, 5% (*w*/*v*) sodium nitrite was added, and after 5 min 10% (*w*/*v*), aluminum chloride was added. After 6 min, 1000 µL of 1 M NaOH and 1200 µL of distilled water were added. The absorbance was read at 510 nm against a blank. A calibration curve was prepared with a (+)-catechin.

TPAs were determined according to the method based on the reaction with an acid-butanol solution [27], with some changes. In brief, 500 µL of GP extract was mixed with 5000 µL of ferrous sulfate solution prepared by dissolving FeSO_4_(H_2_O)_7_ in an HCl-butanol solution. The incubation of the tubes was performed at 95 °C for 15 min, followed by cooling and an absorbance measurement at 540 nm in comparison to a blank.

#### 2.6.3. Determination of Antioxidant Activity by DPPH, FRAP, and ABTS Methods

The antioxidant activity of the GP extracts was determined by DPPH, FRAP, and ABTS assays. Measurements were performed in triplicate for all assays and final results were expressed in Trolox equivalents per dry basis of GP (mg_T_/g_db_).

The DPPH assay was performed according to the method of Bucić-Kojić et al. [28]. In brief, 0.1 mL of the extract was added to the tubes and mixed with 3.9 mL of ethanol solution of the DPPH radical (0.026 mg_DPPH_/mL). After 30 min of incubation in the dark, the absorbance of the reaction mixture was measured at 515 nm. Absolute ethanol served as a blank.

The ABTS test was completed following the method of Re et al. [29], but with minor modifications. A diluted ABTS•+ radical solution (950 µL) was added to 50 µL of the extracts and the absorbance was measured at 734 nm after 10 min of incubation in the dark. The control sample was prepared in the same way, but ethanol was used instead of the extract. Absolute ethanol served as a blank.

The FRAP assay was performed according to the method of Benzie and Strain [30], with modifications. Briefly, 2.7 mL of FRAP reagent was mixed with 270 µL of distilled water and 150 µL of GP extract. Incubation was performed in the dark at 37 °C for 40 min, then the absorbance was measured at 592 nm. The blank sample was prepared in the same way, but distilled water was used instead of the extract.

#### 2.6.4. Measurements of Total Organic Carbon and Total Nitrogen

The catalytic combustion oxidation method at 680 °C was applied for TOC and TN analyses, and catalytically aided combustion oxidation at 900 °C for TC analysis by a TOC analyzer (TOC-L_CPH/CPN_, SSM 5000A, Shimadzu, Japan).

#### 2.6.5. Measurements of Reducing and Individual Sugars Concentrations

Reducing sugars concentration was determined by the DNS method [31,32]. In brief, 500 µL of the extract and DNS reagent were mixed with a vortex and incubated at 100 °C for 5 min. The contents of the test tube were then chilled to ambient temperature and used for an absorbance measurement at 540 nm. The blank sample was made with distilled water instead of the extract.

HPLC with a refractive index detector (RID) was used to analyze the individual sugar contents. The sucrose content was determined using an Aminex^®^ HPX column (HPX-87H, 300 × 7.8 mm, Bio-Rad Laboratories, Hercules, CA, USA). The method was performed with a mobile phase of 5 mM sulfuric acid and a flow rate of 0.6 mL/min at 40 °C for 60 min. The contents of glucose, fructose, arabinose, and sucrose were determined by the Nucleogel^®^ Sugar Pb column (VA, 300 × 7.8 mm, Macherey-Nagel GmbH & Co. KG, Dueren, Germany), with HPLC-grade water as the mobile phase and a flow rate of 0.4 mL/min at 80 °C for 20 min.

#### 2.6.6. Determination of Dry Matter, Ash, Crude Protein, and Free Fats

A fast moisture analyzer (HR-73, Mettler Toledo, Zürich, Switzerland) was used for dry matter content determination. Drying conditions were established by the thermogravimetric method as follows: the standard method involved drying at a temperature of 105 °C, and process termination criterion (switch-off 3: weight loss of 1 mg in 50 s).

The ash content was assessed through total combustion of the samples. Samples weighing 2 g were placed in crucibles and in a muffle furnace heated at 600 °C for 4 h, according to the AACC-08-03 method [33].

The Kjeldahl method was used to determine the crude protein content [34]. The weighted sample (0.2 g ± 0.1 mg), Na_2_SO_4_ (10 g), and CuSO_4_ (0.1 g) were mixed with 15 mL of concentrated H_2_SO_4_ in a glass tube. The combustion was performed at 420 °C for approximately one hour. After combustion, the samples were cooled, mixed with 75 mL of water, and distilled. The percentages of nitrogen (N) and crude protein content (N *×* 6.25) were calculated.

The Soxhlet standard method [35] was applied for free fats determination by the Universal Extraction System (Büchi B-811 LSV, Flawil, Switzerland). Two grams of the sample were used for extraction with n-hexane for three hours. Free fats were collected in glass flasks that had previously been dried and weighed.

#### 2.6.7. Crude Fiber Content Determination

NDF, ADF, and ADL were calculated using a fiber analyzer (FIWE 3, VELP Scientifi-ca, Usmate Velate, Italy), according to the Van Soest technique [36]. The sample was weighed (1.0000 ± 0.0001 g), placed in dried and chilled wells, and then positioned on a fiber analysis device. For NDF analysis, a few drops of n-octanol, 100 mL of NDF solution, and 0.5 g of Na_2_SO_3_ were added. For ADF analysis, a few drops of n-octanol and 100 mL of ADF solution were added. After the samples were brought to a boil, they were refluxed for 60 min, filtered, and purified with cold acetone and boiling water.

The samples were dried at 105 °C for 8 h and weighed. Prior to performing the ADL analysis, ADF analysis was carried out. The flasks containing the filtered samples were cleaned with cold acetone and boiling water before being filled with 25 mL of 72% H_2_SO_4_. The materials were then subjected to a three-hour cold extraction, with stirring every hour. Then, the samples were washed with boiling water until the acidic reaction was removed. Drying (105 °C, 8 h), cooling, and weighing followed. After the sample bottles were burned at 550 °C in a muffle furnace for two hours, cooled, and then weighed in a desiccator, the NDF, ADF, and ADL were computed and adjusted for ash. The difference between NDF and ADF was used to determine the hemicellulose content, while the difference between ADF and ADL was used to calculate the cellulose content. ADL stands for the lignin content.

### 2.7. Statistical Analysis

The statistical data processing program utilized was TIBCO Statistica 14.0.0.15 (TIBCO Software Inc., Palo Alto, CA, USA). A one-way analysis of variance (ANOVA) was conducted to determine the significance of the difference between the arithmetic means of the samples that represented the populations. This was followed by a post hoc test, such as Duncan’s test for multiple ranges (*p* < 0.05).

Principal component analysis (PCA) was determined by selecting the two highest principal components (PC1 and PC2) that divided the samples according to lignolytic (laccase and MnP) and hydrolytic (*β*-glucosidase, xylanase, cellulase, and invertase) enzyme activities; antioxidant activity measured by DPPH, FRAP, and ABTS methods; total phenolic compounds (TPs); total flavonoids (TFs); total proanthocyanidins (TPAs); biomass concentration (BC); carbon and nitrogen ratio (C:N); and duration of SSF (5, 10, and 15 days) in laboratory jars and the tray bioreactor.

The mean values of each phenolic content were compared between day “0” (the untreated sample) and the fermentation day, when the maximal yield of phenolic compounds was reached, using a Student’s *t*-test at a 95% significant level (*p* < 0.05).

## 3. Results and Discussion

In this study, *T. versicolor* was used for the biological treatment of GP for 15 days in laboratory jars and on a larger scale in a tray bioreactor to develop stable conditions for the implementation of the SSF process aimed at improving the extractability of phenolic compounds and the nutritional value of GP. Substrate properties, such as particle size and chemical composition, have a major influence on the performance of the SSF process, as do numerous parameters, such as bioreactor design, moisture content, temperature, availability of nutrients, and fermentation time [37]. Since SSF is a very complex process, the biggest challenge is to scale it up. To transfer the process from laboratory jars to a tray bioreactor, the inoculum must be prepared in a quantity that corresponds to the larger scale of the fermentation. In this case, mycelium cultivated on lignocellulosic materials is usually used as an inoculum, in contrast to experiments in laboratory jars where mycelial plugs from Petri dishes were used. The design and arrangement of the plates in the bioreactor should provide a maximum surface area for contact with the air, but also allow easy handling of the substrate.

The moisture contents of the substrate before the start of SSF in jars and bioreactor were 71.8% and 66.9%, respectively, and decreased over time (Table 3). Maintaining optimum substrate humidity is essential for the growth of microorganisms. In larger systems, drying of the substrate can occur, which was the reason for the difference in the moisture content of the material in the jars and in the bioreactor in this study.

The SSF resulted in a loss of substrate mass of 38.52% after 15 days in the jars, as shown in Table 3. The basidiomycete *T. versicolor* is a filamentous fungus that belongs to white-rot fungi and is known for its ability to degrade the complex structure of lignocellulosic biomass, thanks to a complex system of enzymes it produces during its growth [15]. This leads to the release of high-value components bound to the lignocellulosic matrix, such as bioactive substances, and at the same time to a loss of substrate mass, which contributes to the zero-waste approach.

The temperature in the bioreactor was in the range of 27.0–27.6 °C and the temperature of the substrate on the plates was in the range of 26.7–27.7 °C during the 1–5, 10, and 15 days of fermentation (Table 3). *T. versicolor* can be cultivated on different substrates in a wide temperature range, even between 15 and 32 °C [38,39], but it is important that the temperature is uniform during the particular SSF process.

The part of the results contained in this article was presented at the conference “4th International Conference for Bioresource Technology for Bioenergy, Bioproducts & Environmental Sustainability” (2023).

### 3.1. Enzyme Activity Measurement

In this study, the activities of lignolytic enzymes (laccase and manganese peroxidase (MnP)) and hydrolytic enzymes (*β*-glucosidase, xylanase, cellulase, and invertase) were measured during SSF processes in jars and the tray bioreactor (1–10 and 15 days of SSF).

**Figure 1:**
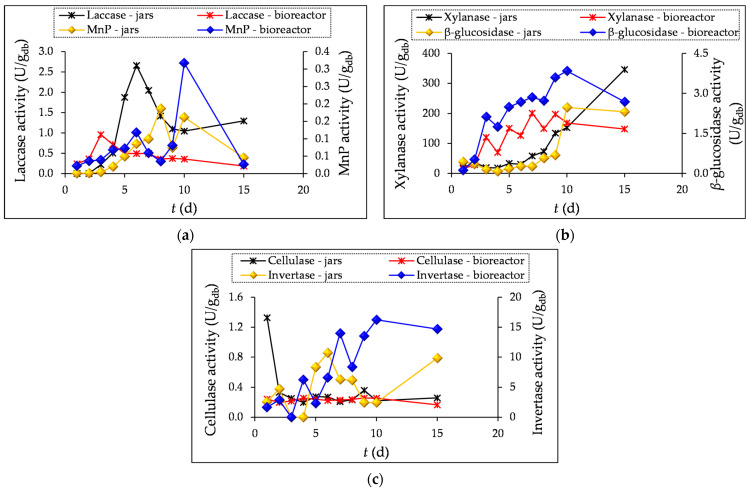
Enzyme activity of lignolytic enzymes laccase and manganese peroxidase (MnP) (**a**), hydrolytic enzymes xylanase and *β*-glucosidase (**b**), and cellulase and invertase (**c**) during SSF of GP (1–10 and 15 days) by *T. versicolor* in laboratory jars and the tray bioreactor.

Of the lignolytic enzymes, the highest activity was observed for laccase (2.66 U/g_db_ in jars (6th day) and 0.96 U/g_db_ in the bioreactor (3rd day)), and of hydrolytic enzymes, for xylanase (346.04 U/g_db_ in jars (15th day) and 200.65 U/g_db_ in the bioreactor (7th day)) (Figure 1a–c).

MnP and invertase activities were highest after 10 days of SSF in the bioreactor (0.32 U/g_db_ and 16.24 U/g_db_, respectively). Cellulase activity was highest after the first day of SSF (1.33 U/g_db_) in jars, after which it decreased and followed the same trend until the end of fermentation.

The activity of enzyme *β*-glucosidase reached a constant increase during 10-day fermentation in both the jars and bioreactor, as shown in Figure 1b. On the 15^th^ day of SSF, there was a decrease in *β*-glucosidase activity, which could be due to a lack of nutrients for the microorganism, leading to a decrease in enzyme production. A similar increasing trend was observed for *β*-glucosidase after 12 days of SSF of grape seeds with *M. anka*, which was reported by Zhao et al. [6].

Literature data report that white-rot fungi can efficiently degrade lignocellulosic biomass to carbon dioxide and water because they have the ability to produce extracellular enzymes that catalyze the biochemical reactions involved in the degradation of the lignocellulosic complex [11]. *T. versicolor* produces a complex system of enzymes and is best known for the production of the lignolytic enzyme laccase [15]. Laccase from *T. versicolor* is used for the enzymatic oligomerization and polymerization of phenolic compounds as an alternative to chemical methods, and catalyzes the oxidation of phenolic compounds with the formation of phenoxy radicals and quinones. In the presence of certain reactants, laccase can be involved in the formation of various homomolecular or heteromolecular oligomers or polymers and phenolic, quinonoid, or quinoneimine structures [40]. Besides laccase, *T. versicolor* produces many other enzymes, such as oxidases, peroxidases, reductases, hydrolases, and pectinases, some of which may also be involved in the degradation of lignocellulosic biomass [6,15,41]. Laccase and MnP contribute to the degradation of lignin, allowing better access to cellulose and hemicellulose, while xylanases enable the efficient degradation of hemicellulose, which increases the accessibility of cellulose and improves the yield of fermentable sugars, which microorganisms can use as a source of carbon and energy. *β*-glucosidase, like other cellulases, are of great importance for cellulose degradation, converting cellulose oligosaccharides into glucose, while invertase catalyzes the hydrolysis of sucrose into glucose and fructose [15,42,43].

Overall, the activity of lignolytic and hydrolytic enzymes is crucial for the success of the SSF process. It improves substrate degradation and increases the yield of the production of a wide range of bioproducts.

### 3.2. Determination of Biomass Concentration and pH Measurement

The main sterol found in filamentous fungi’s cell membrane is ergosterol. The concentration of ergosterol was determined during 1–5, 10, and 15 days of SSF.

**Figure 2:**
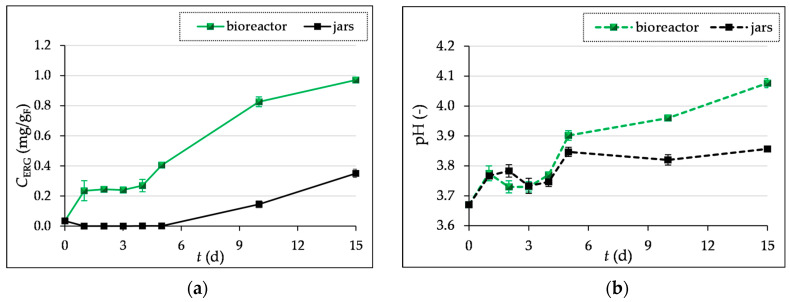
Ergosterol concentration (*C*_ERG_) (**a**) and pH values (**b**) in grape pomace before (day “0”) and during the 1–5-, 10-, and 15-day SSF processes by *T. versicolor* in laboratory jars and the tray bioreactor.

The maximum ergosterol level was found after 15 days of SSF in laboratory jars (0.34 mg/g_F_) and in the tray bioreactor (0.97 mg/g_F_). Figure 2a illustrates how, during the first seven days of SSF in jars, the ergosterol content in GP is very low, and then gradually increases until the end of fermentation. During the SSF in the bioreactor, the ergosterol content is slightly higher in the first four days than in the laboratory jars. The reason for this is the inoculation of the substrate in the bioreactor with the *T. versicolor* already cultivated in the laboratory jars. In comparison to the growth in laboratory jars, where the inoculum was a prepared spore suspension and the culture required more time to adapt to the given growth conditions, the increase in ergosterol content that was visible after the fourth day of fermentation in the bioreactor may be related to the faster adaptation and growth of the already-grown culture.

In addition to monitoring the biomass concentration, the change in the pH value of the substrate during fermentation was also monitored, as the pH value influences the development of the SSF process, i.e., the production of secondary metabolites [37].

Figure 2b shows a decrease in the pH value of the substrate on days 3 and 4 of SSF compared to the first two days of fermentation, which can be explained by the production of organic acids, while after 5 days, a slight increase in pH is observed, which then shows a uniform value with a slight increase until the end of fermentation. The assimilation of organic acids by the microorganism can affect the increase in the pH value [44]. In the bioreactor, the pH value ranged from 3.67 (“0” day) to 4.08, and in the laboratory jars up to 3.86, after 15 days of fermentation.

### 3.3. Chemical Composition of Grape Pomace

The chemical composition of GP can vary greatly, depending on the content of the individual components (seeds, skin, and pulp), the variety, the degree of ripeness, the harvest, and even the winemaking conditions [10,13]. In this study, the results obtained after the chemical composition analysis refer to the dry mass of the sample, and all measurements were made in triplicate.

#### 3.3.1. Determination of Ash, Crude Proteins, and Free Fats Content

According to literature, the ash content in GP is in the range of 3–9.3%, the protein content in the range of 7–14.41%, and the fat content in the range of 6.44–10.47% [8,10,11,45,46].

**Figure 3:**
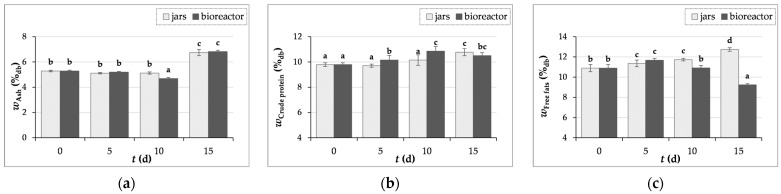
The contents (*w*) of ash (**a**), crude protein (**b**), and free fats (**c**) GP samples before (day “0”) and after 5, 10, and 15 days of SSF with *T. versicolor* in laboratory jars and the tray bioreactor. Samples denoted by distinct lowercase alphabetic letters exhibit statistically significant differences from one another (*p* < 0.05, as determined by the post hoc Duncan’s multiple range test).

In this study, ash content increased by 28% and by 29% after 15 days of fermentation, with *T. versicolor* in laboratory jars and the tray bioreactor (Figure 3a). Crude protein content increased by 10% after 15 days of SSF in jars, while in the bioreactor, the protein content increased by 11% after 10 days of fermentation (Figure 3b). A 17% increase in free fats content was also observed after 15 days of fermentation in jars, while in the bioreactor, free fats content increased by 7% after 5 days of SSF (Figure 3c). From the results presented, it appears that the SSF of GP by *T. versicolor* can improve the nutritional value in terms of mineral, protein, and fatty acid enrichment. An improvement in the nutritional composition of grape seeds after SSF with *Aspergillus niger* was also published by Altop et al. [47].

Gungor et al. [48] reported that the fermentation of GP (enriched with a nutrient solution containing glucose, urea, (NH_4_)_2_SO_4_, peptone, KH_2_PO_4_, and MgSO_4_) with *Aspergillus niger* increased ash content from 4.1% to 8.5% and crude protein content from 12.6% to 28.3%. The production of enzymes and the development of the mycelium of the microorganism used may be linked with the rise in the content of crude protein [47,48]. The results of Abid et al. [11] show an increase in crude protein content from 10.3% to 12.8% and 12.9% after four and eight days of SSF of GP with *Pleurotus cornucopiae* and *Ganoderma resinaceum*, while ash content also increased from 9.3% to 14.8% and 16.2%.

GP contains minerals of which potassium, phosphorus, calcium, and iron are the most abundant. However, the increase in the content of ash and lipids may be related to the growth of the fungus, as the cell wall of fungi contains both lipids and inorganic components and the ash content of fungi varies depending on the fungal species and growth conditions [49,50,51].

Mostafa et al. [38] investigated the composition of wild *T. versicolor* collected from different locations in northern India and reported that this fungus can accumulate metal element from the environment in which it grows, and found the presence of eight metals (Cd, Cr, Cu, Fe, Mn, Zn, Ni, and Co). Fluctuations in ash content are caused by various metal elements in the substrate that have been absorbed by the fungal mycelium and then transferred to the upper body parts of *T. versicolor*. Mostafa et al. [38] also reported that *T. versicolor* has an average content of protein of 8.12–11.06%, fat of 0.93–1.26%, and total ash of 2.42–3.48%.

#### 3.3.2. Crude Fiber Content Determination

**Figure 4:**
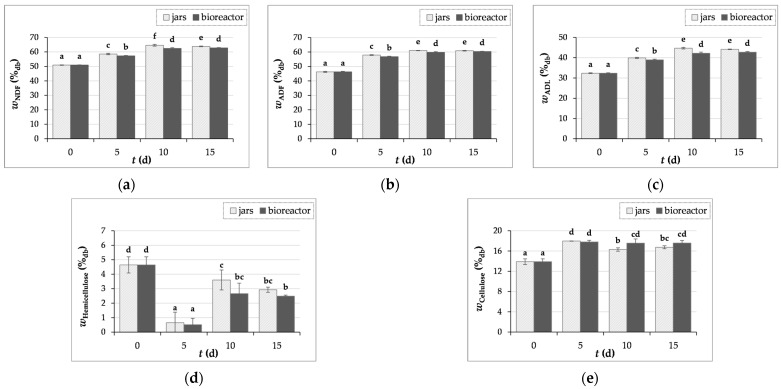
The content of NDFs—neutral detergent fibers (**a**), ADFs—acid detergent fibers (**b**), ADL—acid detergent lignin. Lignin content (**c**), hemicellulose (**d**), and cellulose (**e**) in GP before (day “0”) and after 5, 10, and 15 days of SSF with *T. versicolor* in laboratory jars and the tray bioreactor. Samples denoted by distinct lowercase alphabetic letters exhibit statistically significant differences from one another (*p* < 0.05, as determined by the post hoc Duncan’s multiple range test).

Figure 4a–e show the results of NDF, ADF, and ADL, as well as the cellulose, hemicellulose, and lignin content, which were calculated as written in Section 2.6.7.

During the SSF of GP with *T. versicolor*, an increase in crude fiber content was observed. The NDF, ADF, and ADL contents increased by 27%, 32%, and 38% after 10 days of SSF in jars, and by 23%, 30%, and 32% after 15 days of SSF in the bioreactor, respectively. Similar values for fiber content in the initial GP sample of the Cabernet Sauvignon grape variety were published by Martinović et al. [8], where NDF, ADF, and ADL contents were 50.33 ± 1.69%, 40.03 ± 2.35%, and 25.80 ± 0.92%, respectively.

It is recorded in the literature that the proportion of lignin in GP varies in the range of 11.6–41.3% [52,53,54]. The high lignin content makes the degradation of GP more difficult, as it is more difficult for microorganisms to access cellulose and hemicellulose [54]. In this study, the lignin content in the initial sample of GP was 32.36 ± 0.20%. An increase in the lignin content was observed from 32.36 ± 0.20% to 44.71 ± 0.39% in jars (day 10) and 42.71 ± 0.31% in the bioreactor (day 15) (Figure 4c). Cellulose content in GP increased by 29% and by 28% after 5 days of biological treatment in jars and the bioreactor, respectively, after which the cellulose content decreases until the 15^th^ day of fermentation (Figure 4e). The hemicellulose content decreased (Figure 4d), which may have been caused by the activity of hydrolytic enzymes, such as xylanase, which *T. versicolor* produces. As evidenced by the rise in the concentrations of arabinose and xylose released from the hemicellulose structure during the SSF process conducted in jars and the bioreactor, xylanases act on the breakdown of xylan, the primary unit of hemicellulose [42].

Abid et al. [11] reported that, in GP treated with *Pleurotus cornucopiae* and *Ganoderma resinaceum*, the crude fiber content decreases after four and eight weeks of fermentation, suggesting that the timing of fermentation is crucial and it is essential to determine the optimal lasting effect of SSF depending on the desired product.

The results of crude fiber obtained in our study could be related to the fact that microorganisms initially use simple and readily available carbon sources, such as simple sugars, for their growth. They then produce hydrolytic enzymes that catalyze the hydrolysis reaction of complex polysaccharides into simpler polysaccharides. Therefore, the fungus may be the cause of the increase in lignin and cellulose contents by first consuming other substrate components.

#### 3.3.3. Measurements of Reducing and Individual Sugars Concentrations

From a total of 13 analyzed standards of individual sugars (ribose, cellobiose, galactose, maltose monohydrate, mannose, arabinose, maltotriose, rhamnose, sucrose, glucose, fructose, arabinose, and xylose), the last five sugars were quantified in GP extracts.

**Figure 5:**
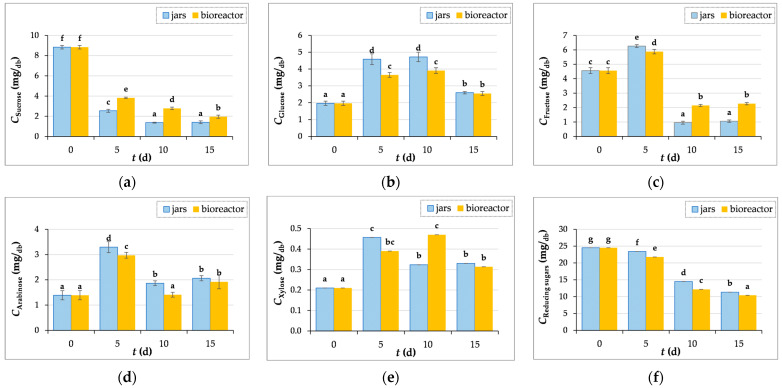
The contents of sucrose (**a**), glucose (**b**), fructose (**c**), arabinose (**d**), xylose (**e**), and reducing sugars (**f**) in GP extracts before (day “0”) and after 5, 10, and 15 days of SSF with *T. versicolor* in laboratory jars and the tray bioreactor. Samples denoted by distinct lowercase alphabetic letters exhibit statistically significant differences from one another (*p* < 0.05, as determined by the post hoc Duncan’s multiple range test).

Figure 5a shows the sucrose content in GP during biological treatment. It is obvious that the content decreases until the end of fermentation, which is due to the presence of the enzyme invertase, which catalyzes the hydrolysis of sucrose into glucose and fructose. The glucose content in the control sample (day “0”) was 1.97 ± 0.13 mg/g_db_, then increased during the 10-day biological treatment in jars (4.72 ± 0.28 mg/g_db_) and bioreactor (3.91 ± 0.17 mg/g_db_), followed by a reduction in the content after 15 days of SSF in jars (2.60 ± 0.07 mg/g_db_) and the bioreactor (2.55 ± 0.12 mg/g_db_) (Figure 5b). The fructose content increases after 5 days of fermentation, and then decreases until the 15^th^ day of fermentation (Figure 5c). The contents of xylose and arabinose increased after SSF both in the jars and in the bioreactor (Figure 5d,e).

It should nevertheless be mentioned that the microbes use simple sugars as an energy source for growth and development at the same time, which accounts for a decrease in certain sugar concentrations during SSF [55]. This is also confirmed by the decrease in the concentration of reducing sugars from 24.52 ± 0.33 mg/g_db_ (“0” day) to 11.34 ± 0.25 mg/g_db_ in the process carried out in jars, and to 10.42 ± 0.14 mg/g_db_ in the bioreactor after 15 days of fermentation (Figure 5f). When the readily available sugars are consumed, the fungi degrade more complex molecules in the lignocellulosic structure to obtain nutrients.

#### 3.3.4. Measurements of Carbon and Nitrogen Content

When analyzing the control samples of GP (day “0”) and after SSF (days 5, 10, and 15), no inorganic carbon (IC_gp_) was detected, so the TOC_gp_ is equal to the total carbon (TC_gp_), as the TOC_gp_ value is calculated from the difference between TC_gp_ and IC_gp_.

**Figure 6:**
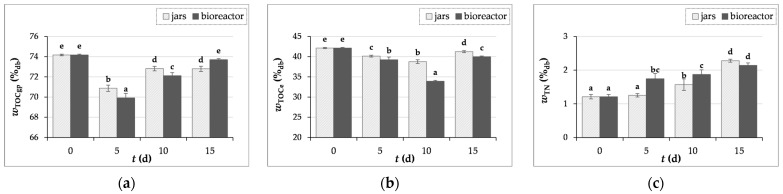
The content (*w*) of total organic carbon in crude samples of GP (TOCgp) (**a**), total organic carbon in GP extract (TOCe) (**b**) and total nitrogen (TN) (**c**) before (day “0”) and after 5, 10, and 15 days of SSF with *T. versicolor* in laboratory jars and the tray bioreactor. Samples denoted by distinct lowercase alphabetic letters exhibit statistically significant differences from one another (*p* < 0.05, as determined by the post hoc Duncan’s multiple range test).

The TOC_gp_ (total organic carbon in solid samples of GP) and TOC_gpe_ (total organic carbon in GP extract) results shown in Figure 6a,b indicate the consumption of organic carbon by microorganisms during the SSF process. Furthermore, carbon is continuously consumed during the SSF process, but it is also released in certain ways, such as glucose, through the action of hydrolytic enzymes, particularly cellulases [56]. The results for the crude protein content, whose rise may be the cause of the increase in total nitrogen, are comparable with the data for TN, which in this work decreased after the first five days of fermentation and then grew until the fifteenth day (Figure 6c).

From the data for the carbon and nitrogen content during fermentation (Figure 6b,c), the C:N ratio was calculated, which was 35 for the initial GP sample and decreased during fermentation. After 15 days of SSF, the C:N ratios in the jars and in the bioreactor were 18 and 19, respectively. The ratio of carbon and nitrogen is an important parameter in the SSF process, and a C:N ratio below 50 is recommended for the cultivation of basidiomycetes, for example to increase the yield of lignolytic enzymes [55]. If it is necessary to correct the C:N ratio, it is possible to combine the substrate with lignocellulosic biomass that has a different chemical composition, for example GP with wheat bran or olive pomace [9,54].

### 3.4. Phenolic Compound and Antioxidant Activity Measurements

#### 3.4.1. Determination of Total Phenolic Compound and Antioxidant Activity

The contents of total phenolic compounds (TPs), total flavonoids (TFs), and extractable proanthocyanidins (TPAs) were determined in the GP extracts before (“0” day) and after the biological treatment with *T. versicolor* (1–5, 10, and 15 days), and the results are explored in mg/g_db_. For the same samples, antioxidant activity was measured using DPPH, FRAP, and ABTS methods, and the results are expressed in Trolox equivalents (mg_T_/g_db_). The SSF had no positive effect on increasing the yields of TP, TF, and TPA, the contents of which decreased by 76%, 76%, and 83% in the laboratory jars, and by 77%, 83%, and 87% in the tray bioreactor after 15 days of fermentation, respectively (Table 4). Various studies indicate that the reason for the decrease in the total phenolic compounds content could be the enzymatic degradation and polymerization of phenolic compounds released during the growth of microorganisms [57,58].

Antioxidant activity is often related to the concentration and type of phenolic compounds present in a given sample. A higher total polyphenol content usually leads to higher antioxidant activity due to the cumulative effect of the different phenolic compounds. However, this correlation is not always linear, as different phenolic compounds have different antioxidant effects. Some polyphenols are more effective antioxidants than others.

The results of this study show that a similar downward trend is observed in the antioxidant activity results of biologically treated GP with all three methods used (DPPH, ABTS, and FRAP, respectively) by 82%, 82%, and 77% in laboratory jars, and by 83%, 72%, and 84% in the tray bioreactor after 15 days of SSF (Table 4). This can be the result of enzymatic degradation or microbial metabolism [58]. Similar results were obtained after the biological treatment of GP with *Rhizopus oryzae* [9]. Authors Zhao et al. [6] published the results of an increase in TPs, TFs, and antioxidant activities measured by the DPPH and ABTS methods after the fermentation of grape seeds with four different microorganisms (*A. niger* CICC 2214, *A. niger* CICC 41481, *Eurotium cristatum*, and *M. anka*). The capacity to release phenolics from the lignocellulosic structure depends mainly on the microorganism used and the enzymes produced during the fermentation process, since numerous studies claim that various hydrolases have a great influence on the degradation of the cell wall of plant matrices, leading to the release or synthesis of phenolic compounds [6,59]. The release or synthesis of phenolic compounds can have a positive effect on increasing antioxidant activity, since each phenolic compound has a specific antioxidant activity, depending on its chemical structure [41,60]. And the antioxidant activity of phenolic compounds depends not only on their content, but also on the donor proton capacity and the ability to delocalize the electrons of the aromatic ring [6].

#### 3.4.2. Principal Components Analysis

**Figure 7:**
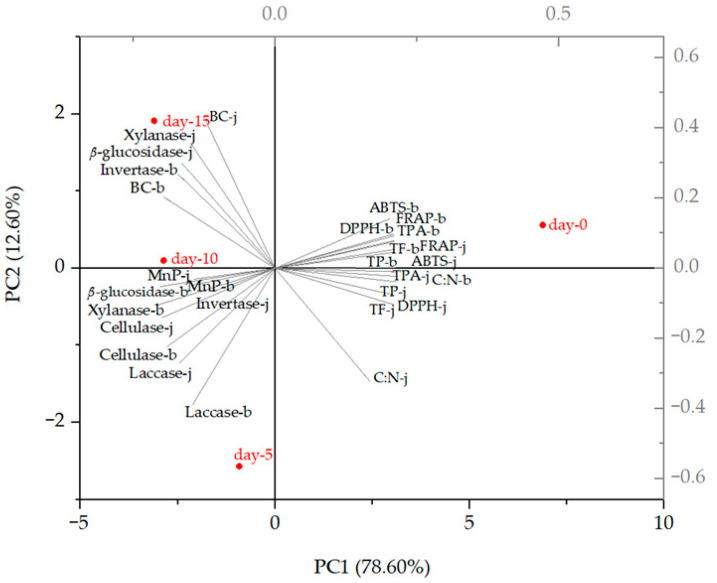
Principal component analysis (PCA) biplot of changes in TPs, TFs, TPAs, BC, C:N, and antioxidant activity measured by DPPH, FRAP, and ABTS methods affected by the activities of lignolytic (laccase and MnP) and hydrolytic (*β*-glucosidase, xylanase, cellulase, and invertase) enzymes after 5, 10, and 15 days of SSF with *T. versicolor* in laboratory jars (j) and the tray bioreactor (b). BC—biomass concentration, C:N—ratio of carbon and nitrogen, TPs—total phenolic compounds, TFs—total flavonoids, and TPAs—total extractable proanthocyanidins.

The PCA biplot showed the changes in TPs, TFs, TPAs, and antioxidant activity measured by DPPH, FRAP, and ABTS methods affected by the activities of lignolytic (laccase and MnP) and hydrolytic (*β*-glucosidase, xylanase, cellulase, and invertase) enzymes after 5, 10, and 15 days of SSF with *T. versicolor* (Figure 7). The two principal components described 91.20% of the total variance in the analyzed data (78.60% for PC1 and 12.60% for PC2). The control group (day “0”) was far from all fermented GP samples, suggesting that the enzyme activities produced during SSF significantly reduced the total phenolic compound content and antioxidant activity, with which they showed a negative correlation. The activities of xylanase and *β*-glucosidase obtained after 15 days of SSF in jars are distributed on the positive side of PC2 and correlate strongly with the biomass concentration (BC). As the biomass concentration increased, the C:N ratio, located on the opposite side of the BC on the biplot, decreased, indicating that *T. versicolor* utilized carbon and nitrogen sources from the substrate during fermentation. Similar results were obtained in the bioreactor, where all enzymes except invertase are on the negative side of PC2.

A look at the biplot shows that there is not much of a difference between the results obtained in the jars and those obtained in the bioreactor in terms of TPs, TFs, TPAs, and antioxidant activity measured by DPPH, FRAP, and ABTS methods.

Although the amount of total phenolic compounds and antioxidant activity decreased during SSF, the fermentation had a positive effect on the increase in certain individual phenolic compounds, the results of which are presented in the next section.

#### 3.4.3. Determination of Individual Phenolic Compound Content

Although phenolic compounds from GP have long been the subject of numerous studies, the growing interest in this area is mainly due to the benefits that these compounds could have for human health. In this study, 21 individual phenolic compounds were identified and quantified in the GP extracts using Ultra-High-Performance Liquid Chromatography (UHPLC). The results indicate that SSF with *T. versicolor* significantly affects the profile and content of phenolic compounds in GP. SSF had a positive effect on the extractability of 13 individual phenolic compounds listed in Table 5. Tian et al. [59] reported that the increase in the content of certain phenolic compounds during SSF may be related to the breakdown of anthocyanins that results in the accumulation of phenolic compounds, which was the case in their research during the SSF of blueberry pomace with fungi (*A. niger*, *A. oryzae*, and *M. anka*) and bacteria (*L. acidophilus*, *L. plantarum*, and *L. casei*).

In this study, the content of phenolic compounds was recorded before (day “0”, *C*_o_) and after SSF (the maximum content of individual phenolic compounds in GP extracts was recorded after a specific day of SSF with *T. versicolor*, *C*_i,max._), as shown in Table 5. No increase in extractability was observed for caffeic acid, ferulic acid, vanillic acid, *p*-coumaric acid, catechin, epicatechin, rutin, or procyanidin B2 after SSF, therefore these results are not presented in the paper. The content of total phenolic compounds in this study after 15 days of SSF was about 12.43 mg/g_db_ and 11.62 mg/g_db_ in jars and in the bioreactor, respectively (as described in Section 3.4.1), while the sum of the maximum content of all 13 quantified individual compounds was about 2.51 mg/g_db_ and 2.84 mg/g_db_. Therefore, the individual quantified phenolic compounds represent only a part of the phenolic compounds present in the obtained extracts, and their content is much lower compared to the total phenolic compounds.

A statistically significant (*p* < 0.05) increase in the extractability of individual phenolic compounds from GP after SSF in laboratory jars was observed for all compounds listed in Table 5, with the exception of gallic acid, syringic acid, and resveratrol. After SSF in a tray bioreactor, a statistically significant (*p* < 0.05) increase in extractability was observed for all compounds listed in Table 5, with the exception of gallic acid, whose extractability decreased significantly after SSF, and syringic acid, whose content was equal to that of the control sample (day “0”). According to the study published by Zhao et al. [6], the content of gallic acid was also significantly reduced after the SSF of grape seeds with the fungi *M. anka* and *E. cristatum*. It has been reported that aromatic compounds can be metabolized by microorganisms by using them as a carbon source via the ring cleavage pathway [61].

Of the hydroxybenzoic acids, a statistically significant increase in extractability was observed for ellagic acid after the first day of fermentation, with a 3.7-fold increase in laboratory jars and a 3.9-fold increase in the tray bioreactor compared to its content in the control sample. The content of *p*-hydroxybenzoic acid increased 1.8-fold in jars (day 10) and 2.2-fold in the bioreactor (day 15), as did the content of 3,4-dihydroxybenzoic acid by 1.7-fold (day 10) in jars and 2.4-fold (day 3) in the bioreactor.

Of the hydroxycinnamic acids, a statistically significant increase in extractability was observed only for *o*-coumaric acid, with an increase of 1.7-fold (day 2) in jars and 1.5-fold (day 1) in the tray bioreactor. SSF with *T. versicolor* also affected the content of flavan-3-ols (epicatechin gallate, and gallocatechin gallate), where the content of epicatechin gallate increased 1.5-fold after SSF in jars and 2.2-fold after SSF in the bioreactor, in both cases after two days of fermentation. The content of gallocatechin gallate increased 1.4-fold and 1.6-fold after two days of fermentation in the jars and bioreactor, respectively.

The positive effect of SSF with *T. versicolor* was also reflected in the flavonols (quercetin and kaempferol), with the maximum increase in quercetin content of 2.9-fold after the first day of fermentation in both processes and kaempferol of 3.3-fold in the jars and 3.5-fold in the bioreactor, also after the first day of fermentation in both cases.

For procyanidin, increased extractability was achieved with procyanidin B1, from 304.27 ± 0.37 µg/g_db_ to 460.39 ± 12.31 µg/g_db_ in jars (day 2) and to 510.34 ± 18.72 µg/g_db_ in the bioreactor (day 1).

In the case of stilbene, a statistically significant 1.2-fold increase in resveratrol was observed after only two days of fermentation in the bioreactor. A statistically significant increase in the yield of *ε*-viniferin was observed in both processes, with the content increasing from 17.52 ± 1.64 µg/g_db_ (“0” day) to 44.33 ± 1.12 µg/g_db_ in jars and to 46.55 ± 1.30 µg/g_db_ in the bioreactor after the first day of fermentation.

Zhao et al. [6] investigated the influence of the SSF of grape seeds with four different microorganisms on the extractability of individual phenolic compounds, with *M. anka* being the most effective, with increases in procyanidin B1, chlorogenic acid, catechin, epicatechin gallate, syringic acid, ferulic acid, and resveratrol contents. Then, *E. cristatum* had an effect on increasing the contents of procyanidin B1, syringic acid, rutin, ferulic acid, and chlorogenic acid, while the two strains of *A. niger* had a minor effect on the release of phenolic compounds.

The literature shows that hydrolases, such as *β*-glucosidase, pectinase, xylanase, and cellulose, are directly related to the release of soluble phenolic compounds from plant matrices. As shown in Section 3.1, the activity of *β*-glucosidase increased consistently during SSF, which may have influenced the release of the aforementioned phenolic compounds [6]. Filamentous fungi can produce phenolic acids as part of their secondary metabolism or release them through the degradation of complex organic compounds, but filamentous fungi also have the ability to degrade phenolic acids through various enzymatic and metabolic processes. Their ability to degrade phenolic compounds can vary depending on the fungal species, available substrates, and growth conditions [41]. Studies also state that *T. versicolor* may contain phenolic acids, such as *p*-hydroxybenzoic acid, protocatechuic acid, and vanillic acid [38].

## 4. Conclusions

The valorization of grape pomace (GP) is an actual topic, not only in the food industry, but also in the cosmetics, biomedicine, and pharmaceutical industries. The use of GP as a substrate under SSF conditions with *T. versicolor* seems to be a potential technique for the release of specific individual phenolic compounds from the lignocellulosic structure, i.e., for the improvement of the profile of bioactive substances and for the general improvement of nutritional and functional properties (increases in mineral, ash, protein, and fat contents) of biologically treated GP. The biological treatment of GP can be crucial for promoting sustainability in viticulture and winemaking, reducing waste and creating additional economic value from by-products that would otherwise be unnecessary or problematic to handle.

## Figures and Tables

**Table 1:** Different types of buffer solutions used for the preparation of GP extracts for enzyme activity measurement.

Lignolytic Enzymes	Buffer Solution	pH
Laccase	50 mM sodium malonate buffer	4.5
Manganese peroxidase (MnP)	50 mM sodium malonate buffer	4.5
**Hydrolytic enzymes**	**Buffer solution**	**pH**
Xylanase	50 mM sodium citrate buffer	5.3
Cellulase	50 mM sodium citrate buffer	4.8
*β*-glucosidase	100 mM sodium acetate buffer	5.0
Invertase	100 mM sodium acetate buffer	4.5

**Table 2:** Extract preparations and methods applied for the analysis of the chemical composition of grape pomace.

Liquid Extract Preparation	Analysis
The mass of 1 g of GP sample (≤1 mm) was weighed into sealed flasks and 40 mL of 50% aqueous ethanol was added. The extraction was conducted in a water bath (Julabo, SW-23, Seelbach, Germany) at 80 °C and 200 rpm for 120 min. Subsequently, the supernatant was collected after centrifugation at 10,000× *g* for 10 min (Z 326 K, Hermle Labortechnik GmbH, Wehingen, Germany) and used for analysis.	Individual phenolic compounds Total phenolic compounds: TPs Total flavonoids: TFs Total extractable proanthocyanidins: TPAs Antioxidant activity: DPPH, FRAP, and ABTS
2. Extracts were obtained by extracting 1 g of GP with 25 mL of distilled water in closed bottles. The extraction was carried out in triplicate in a water bath at 30 °C and 170 rpm for 30 min. Then, the suspension was centrifuged at 10,000× *g* for 10 min and the supernatant was used for analysis.	Total nitrogen (TN) Total organic carbon (TOC_gpe_) * Reducing sugar (RS) Individual sugar
3. For pH measurement, a mass of 2 g of the biologically treated GP was mixed with 10 mL of distilled water, shaken on the vortex for 30 min, and centrifuged at 10,000× g. The pH was measured in the collected supernatant (HI 2211 pH/ORP Meter, Hanna instruments Ltd., Zagreb, Croatia).	pH measurement
**Solid samples of GP**	**Analysis**
GP with a particle size of ≤1 mm was analyzed before (day “0”) and after biological treatment (days 5, 10, and 15).	Dry matter content Ash content Neutral detergent fiber (NDF) Acid detergent fiber (ADF) Acid detergent lignin (ADL) Crude protein Total carbon (TC) Inorganic carbon (IC) Total organic carbon (TOCgp) * Free fat

* TOC_gpe_—total organic carbon in GP extract; TOC_gp_—total organic carbon in solid sample of GP.

**Table 3:** Substrate weight loss and moisture content in laboratory jars, and temperature and moisture content in tray bioreactor during SSF of GP before (day “0”) and after SSF (days 1–5, 10, and 15) with *T. versicolor*.

	Day “0”	Day 1	Day 2	Day 3	Day 4	Day 5	Day 10	Day 15
Weight loss—jars (%_db_)	-	9.0	11.7	14.8	15.6	16.4	25.2	38.5
Moisture content—jars (%_db_)	71.8	71.7	70.5	68.5	65.9	68.4	60.6	51.1
Moisture content—bioreactor (%_db_)	66.9	58.7	57.1	57.2	57.1	49.4	45.4	29.7
*T*_bioreactor_ (°C)	-	27.3	27.0	27.5	27.5	27.6	27.2	27.5
*T*_GP in bioreactor_ (°C)	-	26.7	26.6	27.6	27.7	27.6	27.4	27.3

*T*_bioreactor_—temperature in a tray bioreactor during SSF; *T*_GP in bioreactor_—temperature of the substrate in a tray bioreactor during SSF.

**Table 4:** Total phenolic compounds and antioxidant activity measured by DPPH, ABTS, and FRAP methods in GP extracts before (day “0”) and after SSF (days 1–5, 10, and 15) by *T. versicolor* in laboratory jars and the tray bioreactor.

	Laboratory Jars					
	(mg/g_db_)	(mg/g_db_)	(mg/g_db_)	(mg_T_/g_db_)	(mg_T_/g_db_)	(mg_T_/g_db_)
Day of SSF	TP	TF	TPA	DPPH	ABTS	FRAP
day “0”	50.08 ± 0.08 m	25.14 ± 0.06 n	8.55 ± 0.04 n	57.50 ± 0.00 g	314.00 ± 0.00 k	212.50 ± 0.00 h
day 1	48.95 ± 0.41 l	26.61 ± 0.07 o	7.57 ± 0.06 m	57.00 ± 0.01 fg	206.50 ± 0.00 i	138.50 ± 0.00 g
day 2	41.54 ± 0.01 k	22.21 ± 0.07 m	6.37 ± 0.05 l	53.00 ± 0.00 f	264.00 ± 0.00 j	119.50 ± 0.00 f
day 3	37.47 ± 0.18 j	20.21 ± 0.01 l	5.74 ± 0.04 k	47.00 ± 0.00 e	185.00 ± 0.00 h	114.00 ± 0.00 f
day 4	31.85 ± 0.03 i	16.53 ± 0.04 k	4.45 ± 0.06 j	42.00 ± 0.00 d	167.50 ± 0.01 g	83.50 ± 0.00 e
day 5	23.68 ± 0.11 h	13.91 ± 0.03 j	3.32 ± 0.04 i	27.50 ± 0.00 c	114.00 ± 0.01 f	67.50 ± 0.01 d
day 10	14.79 ± 0.28 e	8.23 ± 0.06 g	1.80 ± 0.03 f	18.50 ± 0.00 b	85.50 ± 0.00 d	58.50 ± 0.01 c
day 15	12.43 ± 0.07 b	5.99 ± 0.03 d	1.44 ± 0.03 d	10.50 ± 0.00 a	57.50 ± 0.00 a	48.50 ± 0.00 b
	**Tray bioreactor**					
	(mg/g_db_)	(mg/g_db_)	(mg/g_db_)	(mg_T_/g_db_)	(mg_T_/g_db_)	(mg_T_/g_db_)
	TP	TF	TPA	DPPH	ABTS	FRAP
day “0”	50.08 ± 0.08 m	25.14 ± 0.06 n	8.55 ± 0.04 n	57.50 ± 0.00 g	314.00 ± 0.00 k	212.50 ± 0.00 h
day 1	22.62 ± 0.11 g	12.63 ± 0.04 i	2.84 ± 0.04 h	56.50 ± 0.00 fg	118.00 ± 0.00 f	67.50 ± 0.00 d
day 2	20.92 ± 0.04 f	11.32 ± 0.04 h	2.57 ± 0.06 g	55.50 ± 0.00 fg	105.00 ± 0.00 e	57.50 ± 0.00 c
day 3	13.30 ± 0.14 c	5.79 ± 0.04 c	1.22 ± 0.04 b	10.50 ± 0.00 a	59.50 ± 0.00 a	34.00 ± 0.01 a
day 4	13.19 ± 0.02 c	6.17 ± 0.06 e	1.34 ± 0.04 c	10.50 ± 0.00 a	78.00 ± 0.00 c	32.00 ± 0.00 a
day 5	14.31 ± 0.04 d	6.91 ± 0.04 f	1.55 ± 0.03 e	11.50 ± 0.00 a	72.00 ± 0.00 b	37.50 ± 0.00 a
day 10	12.64 ± 0.06 b	4.52 ± 0.03 b	1.32 ± 0.02 c	10.50 ± 0.00 a	102.50 ± 0.00 e	35.00 ± 0.00 a
day 15	11.62 ± 0.07 a	4.22 ± 0.07 a	1.09 ± 0.04 a	10.0 ± 0.00 a	88.50 ± 0.00 d	34.00 ± 0.00 a

Each result is expressed as mean (*n* = 3) ± SD. The samples from the population with the lowest mean value of each component are labeled with the letter “a”. Values in the same column labeled with different lowercase letters of the alphabet are statistically significantly different from each other (*p* < 0.05, post hoc Duncan multiple range test); TPs—total phenolic compounds, TFs—total flavonoids, and TPAs—total extractable proanthocyanidins.

**Table 5:** The content of individual phenolic compounds in extracts obtained from grape pomace before SSF (day “0”, *C*_o_) and after SSF by *T. versicolor* in laboratory jars and in the tray bioreactor (maximum content of individual phenolic compounds in grape pomace extracts recorded after a certain duration of SSF, *C*_i,max._).

Phenolic Compound	Day “0”	SSF in Laboratory Jars			SSF in Tray Bioreactor		
	*C*o (µg/gdb) *	*C*i,max. (µg/gdb) *	*p* **	*t*SSF (d)	*C*i,max. (µg/gdb) *	*p* **	*t*SSF (d)
**Phenolic acids (hydroxybenzoic acids)**							
GA	267.77 ± 11.78	275.59 ± 11.90	0.6249	1.	248.40 ± 4.41	0.0450	1.
EA	34.65 ± 3.66	129.11 ± 14.82	0.0125	1.	136.02 ± 3.84	0.0000	1.
*p*-HBA	5.05 ± 2.15	9.33 ± 0.02	0.0007	10.	10.96 ± 0.43	0.0040	15.
SA	86.37 ± 2.15	95.79 ± 3.22	0.0933	10.	86.64 ± 2.86	0.5661	3.
3,4-DHBA	138.61 ± 9.87	237.46 ± 5.73	0.0082	10.	338.03 ± 2.19	0.0005	3.
**Phenolic acid (hydroxycinnamic acid)**							
*o*-CoA	4.43 ± 0.11	7.74 ± 0.33	0.0082	10.	6.83 ± 0.58	0.0263	4.
**Flavan-3-ols**							
EPG	166.69 ± 8.42	246.27 ± 7.32	0.0128	2.	373.05 ± 4.25	0.0001	2.
GCG	291.57 ± 2.35	408.79 ± 15.58	0.0042	2.	480.89 ± 4.18	0.0000	2.
**Flavonols**							
QU	173.32 ± 16.54	504.31 ± 16.98	0.0034	1.	507.29 ± 31.17	0.0067	1.
KA	10.22 ± 1.06	33.78 ± 0.65	0.0017	1.	35.75 ± 1.09	0.0024	1.
**Procyanidin**							
PB1	304.27 ± 0.37	460.39 ± 12.31	0.0019	2.	510.34 ± 18.72	0.0028	1.
**Stilbenes**							
RES	46.07 ± 3.48	56.65 ± 3.54	0.1204	1.	54.46 ± 0.67	0.0353	2.
VIN	17.52 ± 1.64	44.53 ± 1.12	0.0035	1.	46.55 ± 1.30	0.0034	1.

GA—gallic acid, EA—ellagic acid, *p*-HBA—*p*-hidroxybenzoic acid, SA—syringic acid, 3,4-DHBA—3,4-dihydroxybenzoic acid, *o*-CoA—*o*-coumaric acid, EPG—epicatechin gallate, GCG—gallocatechin gallate, QU—quercetin, KA—kaempferol, PB1—procyanidin B1, RES—resveratrol, and VIN—*ε*-viniferin. * The mean (*n* = 3) ± SD is used to express the results. ** The Student’s *t*-test was used for dependent samples with a 95% confidence level to compare the mean values of the concentration of individual phenolic compounds between day “0” (biologically untreated sample of GP) and the day of fermentation when the maximum yield of individual phenolic compounds was reached (red colored values—statistically significant differences with *p* < 0.05).

## Data Availability

All relevant data are available in the manuscript.
